# Bioethanol Production from Cellulose-Rich Corncob Residue by the Thermotolerant *Saccharomyces cerevisiae* TC-5

**DOI:** 10.3390/jof7070547

**Published:** 2021-07-09

**Authors:** Pinpanit Boonchuay, Charin Techapun, Noppol Leksawasdi, Phisit Seesuriyachan, Prasert Hanmoungjai, Masanori Watanabe, Siraprapa Srisupa, Thanongsak Chaiyaso

**Affiliations:** 1 Division of Biotechnology, Faculty of Agro-Industry, Chiang Mai University, Chiang Mai 50100, Thailand; pinpanit_boonchuay@cmu.ac.th (P.B.); charin.t@cmu.ac.th (C.T.); phisit.s@cmu.ac.th (P.S.); prasert.h@cmu.ac.th (P.H.); Siraprapa_sri@cmu.ac.th (S.S.); 2 Division of Food Engineering, Faculty of Agro-Industry, Chiang Mai University, Chiang Mai 50100, Thailand; noppol.l@cmu.ac.th; 3 Department of Food, Life and Environmental Sciences, Faculty of Agriculture, Yamagata University, Tsuruoka, Yamagata 9978555, Japan; mwata@tds1.tr.yamagata-u.ac.jp

**Keywords:** bioethanol, cellulose-rich corncob, thermotolerant *Saccharomyces cerevisiae* TC-5, simultaneous saccharification and fermentation, thermotolerant yeast

## Abstract

This study aimed to select thermotolerant yeast for bioethanol production from cellulose-rich corncob (CRC) residue. An effective yeast strain was identified as *Saccharomyces cerevisiae* TC-5. Bioethanol production from CRC residue via separate hydrolysis and fermentation (SHF), simultaneous saccharification and fermentation (SSF), and prehydrolysis-SSF (pre-SSF) using this strain were examined at 35–42 °C compared with the use of commercial *S. cerevisiae*. Temperatures up to 40 °C did not affect ethanol production by TC-5. The ethanol concentration obtained via the commercial *S. cerevisiae* decreased with increasing temperatures. The highest bioethanol concentrations obtained via SHF, SSF, and pre-SSF at 35–40 °C of strain TC-5 were not significantly different (20.13–21.64 g/L). The SSF process, with the highest ethanol productivity (0.291 g/L/h), was chosen to study the effect of solid loading at 40 °C. A CRC level of 12.5% (*w*/*v*) via fed-batch SSF resulted in the highest ethanol concentrations of 38.23 g/L. Thereafter, bioethanol production via fed-batch SSF with 12.5% (*w*/*v*) CRC was performed in 5-L bioreactor. The maximum ethanol concentration and ethanol productivity values were 31.96 g/L and 0.222 g/L/h, respectively. The thermotolerant *S. cerevisiae* TC-5 is promising yeast for bioethanol production under elevated temperatures via SSF and the use of second-generation substrates.

## 1. Introduction

Globally, the demand for petroleum-based fuels for industry, agriculture, transportation, and private sectors is sharply increasing. An insufficient fuel supply can be alleviated by using alternative fuels. For example, bioethanol is a liquid biofuel produced from biomass by microbial fermentation of various substrates, such as sugar-based substrate, starchy biomass, and lignocellulosic biomass [1,2,3]. Among them, lignocellulosic biomass, a renewable substrate, is the cheapest and most sustainable substrate for bioethanol production [4,5]. Cellulose-rich corncob (CRC) residue is a solid waste obtained from corncob-xylooligosaccharides (corncob-XO) production. During XO production, most of the xylan in KOH-treated corncob is hydrolyzed by cellulase-free endo-xylanase, whereas the cellulose remains. The CRC residue contains a relatively high cellulose content in the range of 74–80% (*w*/*w*), with 10–13% (*w*/*w*) hemicellulose and 2–6% (*w*/*w*) lignin, making it an attractive substrate for fermentable sugar and bioethanol production [6].

Bioethanol production from lignocellulosic biomass can be categorized into three main processes, namely separate hydrolysis fermentation (SHF), simultaneous saccharification and fermentation (SSF), and prehydrolysis-simultaneous saccharification and fermentation (pre-SSF) [7,8]. During SHF, enzymatic hydrolysis and ethanol fermentation are carried out separately, using two bioreactors. The hydrolysis and fermentation step can be carried out independently at their optimum conditions regarding temperature, pH, and time. However, this process requires long operation periods and complex equipment. The bioethanol production rate of SHF might be limited by the high concentration of initial glucose and cellobiose in the hydrolysate [9,10]. In contrast, in the SSF process, enzymatic hydrolysis and ethanol fermentation are carried out simultaneously in a single bioreactor [11]. Consequently, the overall process time, investment cost, substrate feedback inhibition, and contamination risk are decreased; SSF is therefore preferably employed in bioethanol production from lignocellulosic biomass [12,13]. One of the major limitations of SSF is the operation temperature. The optimal temperature for enzymatic hydrolysis is around 40–55 °C [14], whereas that for fermentation is around 20–35 °C [15]. Thus, screening of thermotolerant ethanolic microorganisms, which can grow at 37–50 °C, is required to overcome these limiting factors [5]. As ethanol-producing yeast, *S. cerevisiae* is outstanding candidate suitable for industrial application. Several desirable characteristics of *S. cerevisiae* have been reported, including generally regarded as safe (GRAS) status, high fermentation rate, genetic tractability, and stress tolerant (robustness) [16,17]. Some strains of *S. cerevisiae* are able to grow and produce ethanol at high temperature. Hence, thermotolerant *S. cerevisiae* receives considerable interest for industrial ethanol production [18].

In this sense, an effective thermotolerant yeast strain that is suitable for second-generation bioethanol production from CRC at high temperatures was searched. The effects of temperature and solid loading on bioethanol production by SHF, SSF, and pre-SSF processes, using the selected thermotolerant yeasts, were investigated. To determine the suitability of the selected thermotolerant yeast in ethanol production at the pilot and the industrial scale, the scale-up of bioethanol production in a 5-L stirred tank bioreactor was investigated. The thermotolerant yeast strain *Saccharomyces cerevisiae* TC-5 could grow well and produce bioethanol via CRC, even under low nutrient and high temperature conditions, indicating minimized production costs. High ethanol yields were achieved by strain TC-5 via the fed-batch SSF at elevated temperatures. Using this thermotolerant yeast under optimal conditions, the overall process time for bioethanol production could be reduced and the production of ethanol increased.

## 2. Materials and Methods

### 2.1. Chemicals and Materials

Analytical-grade *p*-nitrophenyl-β-D-glucopyranoside (*p*NPG), arabinose, dinitrosalicylic acid (DNS), and cellobiose were purchased from Sigma (St. Louis, MO, USA). Glucose was purchased from Ajax FineChem (Australia). Malt extract, peptone (Bacto™ Peptone), and yeast nitrogen base without amino acid were purchased from BD Difco™ (Franklin Lakes, NJ, USA). Xylan (Beechwood) was purchased from Megazyme (Wicklow, Ireland). Trehalose and xylose were purchased from Loba Chemie (Mumbai, India). Yeast extract was purchased from HiMedia (Mumbai, India). Lactose, maltose, sulfuric acid, sucrose, and water (HPLC grade) were purchased from RCI Labscan (Bangkok, Thailand). Ethanol (99.8%) was purchased from a Liquor Distillery Organization (Bangkok, Thailand). All other chemicals used in this study were of analytical grade.

A commercial cellulase cocktail (iKnowZyMe AC cellulase) was purchased from Reach Biotechnology Co. Ltd. (Bangkok, Thailand). Endo-glucanase, total cellulase activity (FPase), β-glucosidase, and xylanase were used at concentrations of commercial enzyme were 1200, 50, 140, and 2100 U/mL, respectively.

The CRC residue was obtained from the corncob-XO production process according to the method described by Boonchuay et al. [6]. The solid CRC residue was recovered by filtration using filter cloth and rinsed with tap water to remove other products. Subsequently, CRC residue was dried at 80 °C in a hot air oven (350i, Schwabach, Memmert, Germany) for 48 h. The cellulose, hemicellulose, and lignin contents of the CRC residue were determined by the TAPPI method and analyzed by the Animal Nutrition Laboratory, Department of Animal and Aquatic Sciences, Faculty of Agriculture, Chiang Mai University, Thailand [19,20]. The CRC residue was either used as substrate for hydrolysate (fermentable sugars) production or for bioethanol production via SSF and pre-SSF processes.

### 2.2. Cellulose-Rich Corncob (CRC) Hydrolysate Production

The reaction mixture for CRC hydrolysate production was composed of 7.5% (*w*/*v*) CRC and 22.04 FPU/g_CRC_ of commercial cellulase cocktail in 0.1 M sodium-citrate buffer pH 5.0. The reaction was conducted at 46 °C with a shaking speed of 150 rpm for 96 h, in accordance with the optimal condition for CRC hydrolysate production that reported by Boonchuay et al. [6] and the manual guide for iKnowZyMe AC cellulase. After 96 h, the CRC hydrolysate was recovered by centrifugation (Z236K, Wehingen, Hermle, Germany) at 6000 rpm (4430× *g*) and 4 °C for 10 min and kept at −20 °C until use. The fermentable sugar (glucose, xylose, and arabinose) concentration of the CRC hydrolysate was determined by high-performance liquid chromatography (HPLC).

### 2.3. Medium and Medium Preparation

#### 2.3.1. Yeast Extract–Malt Extract (YM) Medium

The yeast extract–malt extract (YM) medium was composed of yeast extract 4 g/L, malt extract 10 g/L, and glucose 4 g/L. All components were dissolved in distilled water. The initial pH of YM medium was adjusted to 6.5 with 0.1 N NaOH or 0.1 N HCl and autoclaved at 121 °C for 15 min [21].

#### 2.3.2. Separate Hydrolysis and Fermentation (SHF)-Bioethanol Production Medium

Briefly, (NH_4_)_2_SO_4_ 4 g/L, yeast extract 1 g/L, NH_4_H_2_PO_4_ 1 g/L, and MgSO_4_·7H_2_O 0.1 g/L were dissolved in CRC hydrolysate pH 5.0. This CRC hydrolysate produced under optimal condition contained glucose 54.0 g/L, xylose 14.18 g/L, and arabinose 0.65 g/L. The SHF-bioethanol production medium was autoclaved at 121 °C for 15 min before inoculation [6].

#### 2.3.3. Simultaneous Saccharification and Fermentation (SSF)-Bioethanol Production Medium

Sodium citrate buffer pH 5.0 (0.1 M) supplemented with (NH_4_)_2_SO_4_ 4 g/L, yeast extract 1 g/L, NH_4_H_2_PO_4_ 1 g/L, and MgSO_4_·7H_2_O 0.1 g/L was mixed with 7.5, 10, 12.5, and 15% (*w*/*v*) CRC residue. The SSF bioethanol production medium (pH 5.0) was autoclaved at 121 °C for 15 min. After the medium was cooled down, 22.04 FPU/g_CRC_ of commercial cellulase cocktail and 10% (*v*/*v*) prepared seed culture of yeast were transferred into the medium [6].

### 2.4. Microorganisms and Inoculum Preparation

Commercial active dry yeast (*S. cerevisiae*) was purchased from Danstil, Lallemand Inc. (Fredericia, Denmark), and used as a benchmark strain. This commercial strain demonstrates good tolerance to high fermentation temperatures (26–36 °C) and suitable for application both in batch and semi-continuous fermentations. Thermotolerant yeasts, including isolates SB1, SC10, G3, and TC-5, were obtained from the culture collection of the Division of Biotechnology, Faculty of Agro-Industry, Chiang Mai University, Thailand. Based on their 26S rDNA sequences and sugar assimilation test, isolates SB1, SC10, and G3 were identified as *Candida glabrata*. The accession number of these strains are MN784460, MN784462, and KY618710, respectively. Whereas isolate TC-5 was identified as *S. cerevisiae* with the accession number KY681804 (Figure S1). The isolates were first selected based on their ability to grow and produce bioethanol at 42 °C [22]. Stock cultures were maintained in glycerol (30%, *w*/*w*) at −30 °C until use.

For all yeast inoculum preparations, 1.0 mL of glycerol stock was transferred to a 250-mL Erlenmeyer flask containing 50 mL of YM broth and cultivated in an incubator shaker (LSI-3016R, Gyeonggi, Republic of Korea) at 37 °C with a shaking speed of 200 rpm for 24–48 h until the optical density at 600 nm (OD_600_) = 6.0 was reached. Thereafter, the cultivation broth was centrifuged at 6000 rpm (4430× *g*) and 4 °C for 10 min. The obtained cell pellet was washed twice and resuspended in 50 mL of sterile 0.85% (*w*/*v*) NaCl, and the OD_600_ was adjusted to 6.0 before inoculation. Then, the 10% (*v*/*v*) resuspended yeast cell was inoculated to the bioethanol production medium.

### 2.5. Bioethanol Production in the Laboratory Bottle

All experiments were conducted in 100-mL laboratory bottles (Duran, Mainz, Germany) equipped with an airlock, containing 90 mL of each bioethanol production medium. The experiments conducted under static condition were operated in an incubator (SMART i250, Accuplus, Bangkok, Thailand) and those conducted under shaking were operated at 150 rpm in the incubator shaker (LSI-3016R, Labtech, Gyeonggi, Republic of Korea).

#### 2.5.1. Effects of Temperature and Process on Bioethanol Production

Separate hydrolysis and fermentation (SHF) process. The effects of temperature on bioethanol production via SHF, using the selected thermotolerant yeast *S. cerevisiae* TC-5 and commercial *S. cerevisiae* (control), were determined using SHF-bioethanol production medium. The effect of temperature on bioethanol production of all tested strains was investigated at 35, 37, 40, or 42 °C for 120 h under static and limited O_2_ conditions. Samples were periodically taken at 12-h intervals, and the levels of remaining sugars and ethanol were determined by HPLC.

Simultaneous saccharification and fermentation (SSF) process. After the autoclaved SSF-bioethanol production medium was cooled down, 22.04 FPU/g_CRC_ of commercial cellulase cocktail and 10% (*v*/*v*) of each seed culture of the thermotolerant strain *S. cerevisiae* TC-5 or commercial *S. cerevisiae* (control) were inoculated into the medium. The effect of temperature on bioethanol production of all tested strains was investigated at 35, 37, 40, or 42 °C at 150 rpm for 120 h, under limited O_2_ conditions in the incubator shaker. Samples were periodically taken at 12-h intervals, and the concentrations of residual sugars and ethanol were determined by HPLC.

Prehydrolysis-simultaneous saccharification and fermentation (pre-SSF) process; Bioethanol production via pre-SSF was performed using SSF-bioethanol production medium. Commercial cellulase cocktail (22.04 FPU/g_CRC_) was added to the medium, and the prehydrolysis step was conducted at 46 °C, 150 rpm, for 24 h. After that, 10% (*v*/*v*) of prepared seed culture of the thermotolerant yeast strain TC-5 or commercial *S. cerevisiae* (control) was subsequently inoculated. The effect of temperature on bioethanol production of all tested strains was investigated at 35, 37, 40, or 42 °C, keeping the other parameters identical to those in the SSF process. Samples were periodically taken at 12-h intervals. The concentrations of residual sugars and ethanol in the samples were determined by HPLC. The optimal temperature and fermentation conditions, resulting in the highest ethanol concentration, yield, and productivity, were selected for the following experiment.

#### 2.5.2. Effect of Solid Loading on Bioethanol Production by *S. cerevisiae* TC-5

Batch simultaneous saccharification and fermentation (batch SSF) process. The effect of solid loading on bioethanol production by *S. cerevisiae* TC-5 via the batch SSF process was studied at 7.5, 10, 12.5, and 15% (*w*/*v*) CRC residue, using SSF-ethanol production medium. The experimental conditions were 40 °C, 150 rpm, for 168 h. The inoculum size and enzyme dosage were fixed at 10% (*v*/*v*) and 22.04 FPU/g_CRC_, respectively. Samples were periodically taken at 12-h intervals. The concentration of residual sugar and ethanol was determined by HPLC.

Fed-batch simultaneous saccharification and fermentation (fed-batch SSF) process; Fed-batch SSF experiments with 10, 12.5, and 15% (*w*/*v*) CRC residue were performed by keeping the other parameters constant. According to our previous study [6], the effect of the CRC concentration on hydrolysate production was studied using a statistical design at different levels from 2.5–17.5% (*w*/*v*). The highest fermentable sugar concentration was attained when using the 7.8% (*w*/*v*). However, when the CRC concentration was increased to 17.5% (*w*/*v*), the fermentable sugar yields (g/g_CRC_) was lower than that obtained from other experiments. Whereas, at low substrate loading experiment (2.5–5.0%, *w*/*v*), the glucose and total sugar concentration (g/L) was lower than that of high substrate loading experiment. The initial CRC residue concentration of all experiments was 7.5% (*w*/*v*). The fed-batch SSF reactors were fed with 2.25 g of CRC residue at 48, 72, and 96 h to increase the final substrate loading to 10, 12.5, and 15% (*w*/*v*) CRC residue, respectively. Samples were periodically taken at 12-h intervals. The concentrations of residual sugars and ethanol were determined by HPLC. The optimal solid loading and fermentation conditions, resulting in the maximum bioethanol concentration, yield, and productivity, were then selected for further experiments.

### 2.6. Bioethanol Production via Fed-Batch SSF by S. cerevisiae TC-5 in a Bioreactor

For the scale-up of bioethanol production employing *S. cerevisiae* TC-5, the fed-batch SSF process was performed in a 5-L stirred tank bioreactor (MDFT-N-5L, BE Marubishi, Bangkok, Thailand) with a 3-L working volume. The experiment was performed with 7.5% (*w*/*v*) CRC residue as the initial solid loading. The 75 g of CRC residue was fed after 48 and 72 h to increase the final solid loading to 12.5% (*w*/*v*). Samples were periodically taken at 12-h intervals. The concentration of residual sugar and ethanol was determined by HPLC.

### 2.7. Analytical Methods

#### 2.7.1. Sugar and Ethanol Determination

Samples were centrifuged at 13,000 rpm (16,060× *g*) and 4 °C for 15 min. The obtained supernatant was filtered through a nylon membrane filter (0.2 μm, FiltrEX, USA) and subjected to HPLC analysis (SCL-10Avp, Kyoto, Shimadzu, Japan) with an Aminex HPX 87H column (300 × 7.8 mm; Bio-Rad, Hercules, CA, USA). The mobile phase consisted of 5.0 mM H_2_SO_4_ as an eluent at a flow rate of 0.60 mL/min. The column thermostat was set at 40 °C. Sugar and ethanol were detected using a refractive index (RI) detector (RID-10A, Shimadzu, Kyoto, Japan) over 25 min [6].

#### 2.7.2. Enzyme Assay

Endo-glucanase activity was measured using 0.5% (*w*/*v*) sodium carboxymethyl cellulose solution in 0.1 M sodium-citrate buffer (pH 5.0) as a substrate, according to the modified method of Zhang et al. [23]. Total cellulase activity (FPase; filter paper unit or FPU) was measured using filter paper as substrate, according to the modified method of Ghose [24]. The β-glucosidase activity was measured using *p*-nitrophenyl-β-D-glucopyranoside (*p*NPG) in 0.1 M sodium-citrate buffer (pH 5.0) as substrate and calculated using the molar extinction coefficient, ɛ400 = 18,300 M^−1^cm^−1^, according to the methods of Salma [25] and Boonchuay et al. [6]. Endo-xylanase activity was measured using 1.0% (*w*/*v*) beechwood xylan solution in 0.1 M sodium-citrate (pH 5.0) as substrate [26].

#### 2.7.3. Calculation and Statistical Analysis

All experiments were carried out in triplicate. The data were analyzed for statistical significance using one-way analysis of variance (ANOVA), followed by Duncan’s multiple range test (*p* < 0.05). The statistical software package SPSS v. 17 was used in the analysis of the experimental data. The fermentable sugar yield, the conversion of cellulose to glucose (hydrolysis efficiency, %), the initial glucose in the SSF-bioethanol production medium, the theoretical ethanol yield (Y, %), the conversion of cellulose to ethanol (%), ethanol yield (Y_EtOH_, g/g), and ethanol productivity (Q_p_, g/L/h) were calculated according to Boonchuay et al. [6].

## 3. Results

### 3.1. Composition of Cellulose-Rich Corncob (CRC) Residue and CRC Hydrolysate Production

The cellulose, hemicellulose, and lignin contents of CRC residue were 74.36 ± 1.11%, 18.19 ± 0.90%, and 3.96 ± 0.07%, respectively. The CRC residue was a solid waste obtained from corncob-XO production. After hydrolysis by commercial cellulase cocktails, CRC hydrolysate consisted of glucose 54.04 ± 0.91 g/L, xylose 14.18 ± 0.90 g/L, and arabinose 0.65 ± 0.04 g/L. The hydrolysis efficiency (85.69 ± 2.03%) was calculated, and the produced CRC hydrolysate was further applied as a carbon source for bioethanol production by the thermotolerant yeast via SHF.

### 3.2. Selection of the Thermotolerant Yeast for Bioethanol Production from CRC Hydrolysate

**Figure 1:**
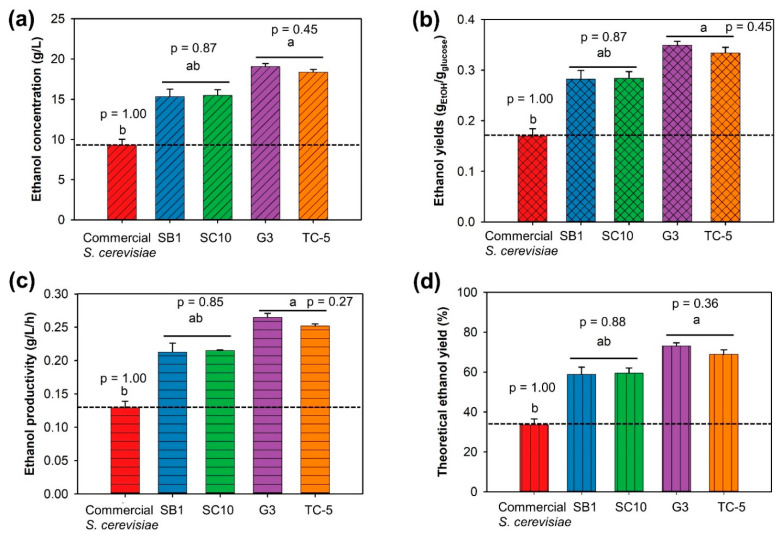
Comparison of ethanol concentration (**a**), ethanol yield (**b**), ethanol productivity (**c**), and theoretical ethanol yield (**d**) from cellulose-rich corncob hydrolysate by four thermotolerant yeasts and commercial *S. cerevisiae* at 42 °C for 72 h. Note: values are presented as mean ± standard deviation. Different letters above the bars denote statistically significant different. Bar chart with the same pattern and letter are not significantly different. The level of significance was tested by Duncan’s multiple range test. (Each kinetic value (ethanol concentration, ethanol yield, ethanol productivity, or theoretical ethanol yield) was compared between yeast strains).

The ability of strain SB1, SC10, G3, and TC-5 to produce bioethanol from CRC hydrolysate was studied at a high temperature (42 °C). Figure 1 shows that *C. glabrata* G3 produced the highest ethanol concentration (C_EtOH_), ethanol productivity (Q_P_), ethanol yield (Y_p/s_), and theoretical ethanol yield (Y) of 19.05 g/L, 0.256 g/L/h, 0.349 g_EtOH_/g_glucose_, and 68.45%, respectively. However, *S. cerevisiae* TC-5 also showed the highest and comparable ethanol concentration, ethanol productivity, ethanol yield, and theoretical ethanol yield of 18.38 g/L, 0.252 g/L/h, 0.334 g_EtOH_/g_glucose_, and 66.05%, respectively. In addition, ethanol production using both of thermotolerant yeast strains G3 and TC-5 were not significantly difference.

### 3.3. Bioethanol Production in the Laboratory Bottle

#### 3.3.1. Effects of Temperature and Processes on Bioethanol Production by *S. cerevisiae* TC-5

**Figure 2:**
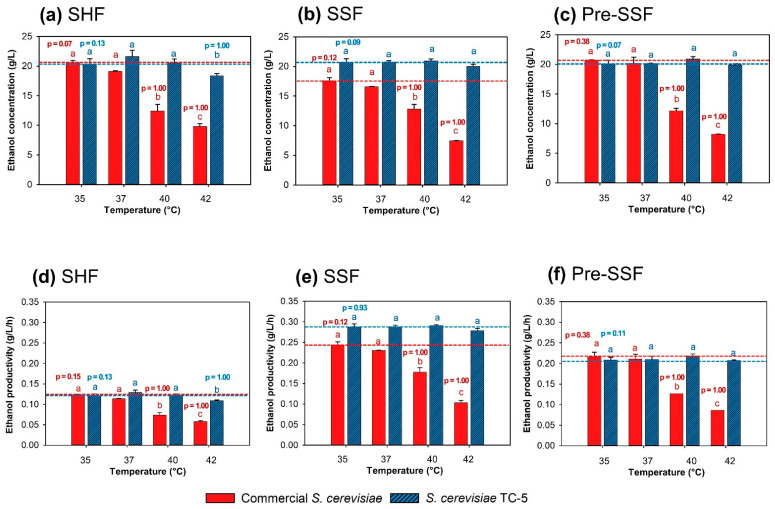
Ethanol production via separate hydrolysis and fermentation (SHF) (**a**), simultaneous saccharification and fermentation (SSF) (**b**), and prehydrolysis-simultaneous saccharification (pre-SSF) (**c**); ethanol productivity via SHF (**d**), SSF (**e**), and pre-SSF (**f**) by commercial *S. cerevisiae* and thermotolerant *S. cerevisiae* TC-5 at 35, 37, 40, and 42 °C for 72 h. Note: values are presented as mean ± standard deviation. Different letters above the bars denote statistically significant different. The level of significance was tested by Duncan’s multiple range test. Bar chart with the same pattern and letter are not significantly different (ethanol concentration or productivity of each yeast strain were compared between temperature).

Separate hydrolysis and fermentation (SHF) process. The effect of temperature on bioethanol production from CRC hydrolysate by *S. cerevisiae* TC-5 and commercial *S. cerevisiae* (control) via SHF was investigated at 35–42 °C. When the temperature was set at 35 °C, glucose was completely consumed by the thermotolerant yeast strain TC-5 and the commercial *S. cerevisiae* (Figures S2 and S3). The ethanol concentration and ethanol yield of both tested strains ranged from 20.33–20.59 g/L and 0.373–0.378 g_EtOH_/g_glucose_, respectively (Figure 2a and Table S1). In addition, the range of theoretical ethanol yield at 35 °C was 78.01–79.01%. The ethanol production at 35 °C of both tested strains did not significantly differ. However, elevated temperature (37–42 °C) had a negative effect on ethanol fermentation by commercial *S. cerevisiae*. At 40 and 42 °C, commercial *S. cerevisiae* could not metabolize glucose completely, although the cultivation period was extended to 120 h (Figure S2). When the temperature ranged from 35 to 42 °C, the ethanol concentration of this strain decreased continuously from 20.59 to 9.81 g/L, theoretical ethanol yield decreased from 79.01 to 37.64%, and ethanol yield dramatically decreased from of 0.378 to 0.180 g_EtOH_/g_glucose_. In contrast, temperatures of 37 and 40 °C did not affect the ethanol production by the thermotolerant yeast strain TC-5. At these temperatures, glucose was rapidly and completely consumed within 48 h. When the SHF process was investigated at 42 °C, the glucose consumption of strain TC-5 was slightly impeded until 72 h of cultivation time (Figure S3). After that, the consumption was steady, and a small amount of glucose remained in the fermentation medium. The significantly highest ethanol concentration of 20.50 g/L was obtained from strain TC-5 when the cultivation temperature was 40 °C (Figure 2a). Moreover, the ethanol productivity of strain TC-5 at 35–40 °C was not significantly different (Figure 2d). However, ethanol concentration (Figure 2a), ethanol productivity (Figure 2d), ethanol yield (Table S1), and theoretical ethanol yield (Table S1) of strain TC-5 slightly decreased when the temperature increased to 42 °C.

Simultaneous saccharification and fermentation (SSF) process; To determine the feasibility to produce bioethanol from CRC by using the thermotolerant TC-5 via SSF at high temperatures, fermentation was carried out at 35, 37, 40, or 42 °C, 150 rpm, under limited O_2_ condition. The effect of temperature on ethanol production was examined and is shown in Figure 2b,e, Figures S4 and S5, and Table S1. At 35 and 37 °C, the released glucose was rapidly consumed within 72 h by commercial *S. cerevisiae* since the sugar consumption by yeast might be equal to the rate of enzymatic hydrolysis; glucose in the fermentation medium was not detected (Figures S4 and S5). The final ethanol concentration of the commercial strain at 35 and 37 °C was 17.58 and 16.58 g/L, respectively. Theoretical ethanol yields of 67.46 and 63.62% were obtained, respectively (Table S1). On the contrary, at 40 and 42 °C, the glucose concentration continuously increased because high temperature might inhibit the ethanol fermentation and growth of the commercial strain. Under high temperatures (40 and 42 °C), the ethanol production of the commercial yeast strain was relatively low at 7.45–12.81 g/L (Figure 2b,e, Table S1). The ethanol yield of commercial *S. cerevisiae* also considerably decreased with increasing temperatures. In the SSF process, the high temperatures at 40–42 °C did not affect the ethanol fermentation of strain TC-5 (Figure 2b,e). The ethanol concentration at 40 °C was 20.92 g/L, with an ethanol yield of 0.384 g_EtOH_/g_glucose_ and an ethanol productivity of 0.291 g/L/h. When the temperature was elevated to 42 °C, strain TC-5 obtained an ethanol concentration, ethanol yield, and ethanol productivity of 20.02 g/L, 0.368 g_EtOH_/g_glucose_, and 0.278 g/L/h, respectively.

Prehydrolysis-simultaneous saccharification and fermentation (pre-SSF) process; The effect of temperature on ethanol production during the pre-SSF process by thermotolerant *S. cerevisiae* TC-5 and commercial *S. cerevisiae* was examined (Figure 2c,f; Table S1). After prehydrolysis at 46 °C for 24 h, the glucose concentration was rapidly increased (~50 g/L). Subsequently, each yeast inoculum was added to the SSF-bioethanol production medium, and the temperature was adjusted to 35, 37, 40, and 42 °C. The glucose consumption of commercial *S. cerevisiae* at 35 and 37 °C increased within 24–72 h (Figure S6). At the same time and under high temperatures (40 and 42 °C), the glucose consumption decreased dramatically. Thereafter, the consumption rate was steady and glucose was not consumed completely, even when the cultivation period was extended to 144 h (Figure S6). When the temperature was increased from 35 to 42 °C, the ethanol concentration and theoretical ethanol yield of commercial *S. cerevisiae* decreased from 20.71 to 8.24 g/L, and from 79.47 to 31.62%, respectively. Glucose was rapidly metabolized by strain TC-5 at 35 and 37 °C. This strain also showed effective glucose use in a temperature range of 35–42 °C (Figure S7). An ethanol concentration in the range of 19.91–20.89 g/L was obtained, with a theoretical ethanol yield of 76.40–80.16% (Table S1).

#### 3.3.2. Effect of Solid Loading on Bioethanol Production by *S. cerevisiae* TC-5

**Figure 3:**
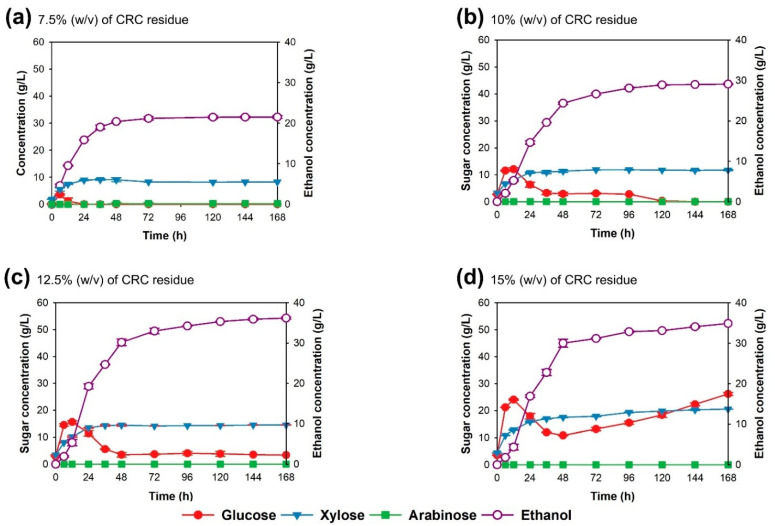
Effect of solid loading at 7.5 (**a**), 10 (**b**), 12.5 (**c**), and 15% (**d**) (*w*/*v*) cellulose-rich corncob residue on ethanol fermentation by the thermotolerant *S. cerevisiae* TC-5 via simultaneous saccharification and fermentation conducted in a 100-mL laboratory bottle at 40 °C for 168 h.

Batch simultaneous saccharification and fermentation (batch SSF) process. The bioethanol production by strain TC-5 via batch SSF was investigated using CRC as substrate at 40 °C, 150 rpm, for 168 h. Four concentrations of solid loading, namely 7.5, 10, 12.5, and 15% (*w*/*v*) CRC, were studied. At 7.5% (*w*/*v*) CRC residue, glucose was rapidly consumed within 12 h as the sugar consumption by yeast might equal the rate of enzymatic hydrolysis (Figure 3a). Therefore, glucose was not detected in the fermentation medium. The ethanol concentration and theoretical ethanol yield were 20.92 g/L and 80.26%, respectively (Table 1). At 10% (*w*/*v*) solid loading, glucose was completely consumed within 120 h (Figure 3b), with ethanol concentration and theoretical ethanol yield values of 28.90 g/L and 88.37%, respectively. However, at 12.5 and 15% (*w*/*v*) CRC loading, glucose was not completely used (Figure 3c,d). The ethanol concentrations were 35.91 and 34.90 g/L, with theoretical ethanol yields of 87.86 and 71.13%, respectively (Table 1).

**Figure 4:**
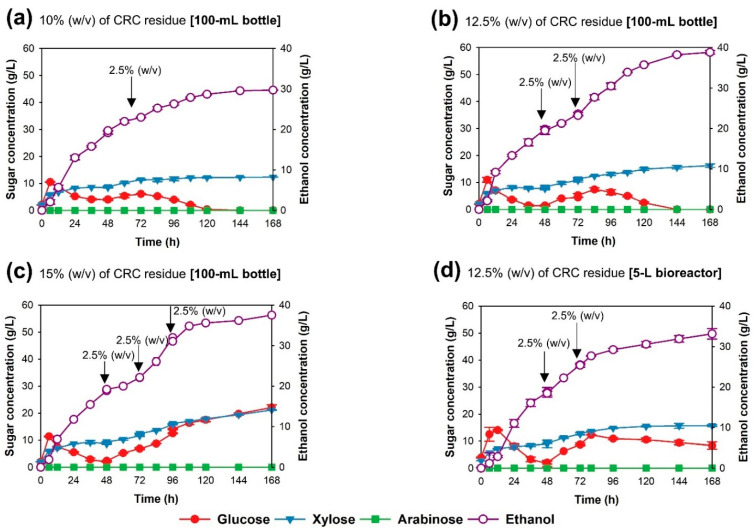
Time course of ethanol production from cellulose-rich corncob residue by the thermotolerant *S. cerevisiae* TC-5 via fed-batch simultaneous saccharification and fermentation, using 10 (**a**), 12.5 (**b**), and 15% (**c**) (*w*/*v*) cellulose-rich corncob residue, conducted in a 100-mL laboratory bottle at 40 °C for 168 h; time course of ethanol production via fed-batch simultaneous saccharification and fermentation using 12.5% (*w*/*v*) cellulose-rich corncob residue by the thermotolerant *S. cerevisiae* TC-5, conducted in a 5-L bioreactor at 40 °C for 168 h (**d**).

Fed-batch simultaneous saccharification and fermentation (fed-batch SSF) process. To investigate the possibility of bioethanol production by *S. cerevisiae* TC-5 via fed-batch SSF process at 40 °C, 150 rpm, for 168 h, fed-batch SSF was performed by using 10, 12.5, and 15% (*w*/*v*) final solid loading. The obtained ethanol concentration, ethanol productivity, ethanol yield, and theoretical ethanol yield are shown in Table 1. As depicted in Figure 4a–c, with a higher CRC solid loading, longer fermentation periods were required. At the CRC solid loading of 10% (*w*/*v*), ethanol concentration, ethanol productivity, and theoretical ethanol yield values of 28.70 g/L, 0.239 g/L/h, and 87.74%, respectively, were achieved at 120 h (Figure 4a and Table 1). At 12.5% (*w*/*v*) CRC solid loading, the highest ethanol concentration and theoretical ethanol yield values of 38.23 g/L and 93.51%, respectively, were obtained within 144 h (Figure 4b and Table 1). Ethanol yield and ethanol productivity were 0.478 g_EtOH_/g_glucose_ and 0.265 g/L/h, respectively. Surprisingly, ethanol production from 12.5% loading via fed-batch SSF process was significantly higher than that from the batch SSF process at 12.5% (*w*/*v*) CRC solid loading (Table 1). At the same solid loading, glucose was accumulated and was not metabolized completely in the batch SSF (Figure 3c), whereas the released glucose was completely used in the fed-batch SSF (Figure 4b). In contrast, bioethanol production via fed-batch SSF with 15% (*w*/*v*) CRC solid loading showed that the sugar concentration continuously increased, and sugar was accumulated in the fermentation medium (Figure 4c). The ethanol concentration was 37.55 g/L, with ethanol yield and theoretical ethanol yield values of 0.391 g_EtOH_/g_glucose_ and 76.56%, respectively (Table 1). However, the values obtained for the fed-batch SSF at 15% (*w*/*v*) CRC solid loading were higher than those obtained for the batch SSF at 15% (*w*/*v*) CRC solid loading (Figure 3d,c, and Table 1).

### 3.4. Bioethanol Production via Fed-Batch Simultaneous Saccharification and Fermentation (Fed-Batch SSF) by S. cerevisiae TC-5 in a Bioreactor

For the scale-up of bioethanol production by *S. cerevisiae* TC-5, fed-batch SSF was performed in a 5-L stirred tank bioreactor with a 3-L working volume. The experiment was performed with the final solid loading of 12.5% (*w*/*v*); the glucose concentration decreased slowly, and glucose remained in the fermentation medium (Figure 4d). An ethanol concentration of 31.96 g/L, with an ethanol yield and a theoretical ethanol yield of 0.400 g_EtOH_/g_glucose_ and 78.20%, respectively, could be obtained.

### 3.5. Mass Balance of Bioethanol Production by the Thermotolerant S. cerevisiae TC-5

**Figure 5:**
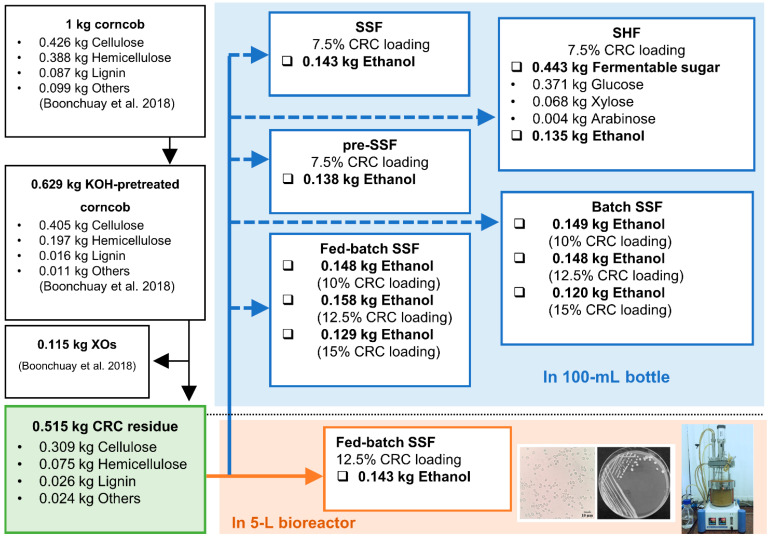
Mass balance of bioethanol production from cellulose-rich corncob residue by the thermotolerant *S. cerevisiae* TC-5.

Figure 5 shows the mass balance for bioethanol production (based on 1 kg of raw corncob). One kg of raw corncob contained 426 g of cellulose (42.6%), 388 g of hemicellulose (38.82%), 87 g of lignin (8.69%), and 99 g of other components (9.88%). After KOH pretreatment, 629 g of treated corncob could be obtained.

The bioethanol production in the laboratory bottle by strain TC-5 via SHF, SSF, and pre-SSF with 7.5% (*w*/*v*) CRC residue was performed at 40 °C. When applying SHF, 443 g of fermentable sugar, 371 g of glucose (83.73%),68 g of xylose (15.26%), and 4 g of arabinose (1.01%) were obtained, with an ethanol yield of 135 g (0.263 g_EtOH_/g_CRC_ or 0.376 g_EtOH_/g_glucose_). The SSF process resulted in the highest ethanol yield of 143 g (0.277 g_EtOH_/g_CRC_ or 0.384 g_EtOH_/g_glucose_). With regard to the pre-SSF process, an ethanol yield of 138 g (0.268 g_EtOH_/g_CRC_ or 0.383 g_EtOH_/g_glucose_) was obtained. Hence, we selected SSF for the bioethanol production in laboratory bottles by strain TC-5 at 40 °C via batch and fed-batch SSF. For batch SSF, the ethanol yields from 10, 12.5 and 15% (*w*/*v*) CRC residue were 149, 148, and 120 g or 0.289, 0.287, and 0.233 g_EtOH_/g_CRC_, respectively. Moreover, the highest ethanol yields of 148, 158, and 129 g or 0.287, 0.306, and 0.250 g_EtOH_/g_CRC_ were obtained from fed-batch SSF with 10, 12.5, and 15% (*w*/*v*) CRC residue. The scaled-up fed batch SSF with 12.5% (*w*/*v*) CRC residue provided a slightly lower ethanol yield of 143 g (0.256 g_EtOH_/g_CRC_ or 0.400 g_EtOH_/g_glucose_) than the bioethanol production in laboratory bottles.

## 4. Discussion

During the XO production process, almost all of the xylan, which is the main hemicellulose found in corncob, was hydrolyzed by endo-xylanase, and released into the liquid phase. In the solid phase of the CRC residue, the cellulose content remained constant [6]. Hence, the cellulose ratio in CRC was relatively high. Cellulose is a polysaccharide composed of linear homopolymers of glucose units linked together by β-1,4 glycosidic bonds [27], making CRC a promising substrate for lignocellulosic hydrolysate (fermentable sugar) production.

The species *C. glabrata* is a thermotolerant ethanol-producing yeast which can tolerate both high temperatures and high acid concentrations [6,28]. However, it is also an opportunistic human pathogen [29]. The species *S. cerevisiae* is the most widely employed yeast in industrial ethanol production because it can tolerate at wide range of pH and temperature levels (30–42 °C). Moreover, *S. cerevisiae* can produce high ethanol yields even under the limited O_2_ condition [15,30,31,32]. Regarding the safety aspect, *S. cerevisiae* is generally regarded as safe (GRAS) and frequently employed, with high potential in the industrial ethanol production [33]. Therefore, the isolated thermotolerant *S. cerevisiae* TC-5 was selected for bioethanol production from CRC residue.

In the SHF process, enzymatic hydrolysis and ethanol fermentation are carried out separately using two separate vessels, making this process time- and labor-consuming [10,11]. These limitations can be overcome by employing the SSF process, which is a combination of both enzymatic hydrolysis and ethanol fermentation in a single bioreactor [34]. Hence, the effect of temperature on bioethanol production from CRC via SSF by the thermotolerant yeast strain TC-5 was also considered. The thermotolerant yeasts strain TC-5 showed satisfactory performance regarding bioethanol production. The glucose was completely consumed at all studied temperatures (35–42 °C), and xylose and arabinose were accumulated. Generally, *S. cerevisiae* can ferment glucose (hexose) into ethanol, but cannot ferment the pentoses, especially xylose and arabinose [15]. This limitation might be overcome by co-fermentation with pentose-fermenting yeasts. However, CRC hydrolysate contains low xylose and arabinose concentrations of only 14.18 and 0.65 g/L, respectively. A higher concentration of xylose has been detected in hydrolysate produced from various types of lignocellulosic biomass e.g., sugarcane bagasse (56 g/L), rice straw (44 g/L), and wheat straw (25 g/L) [35,36,37]. Generally, almost of pentose-fermenting yeasts cannot grow under high ethanol and inhibitor concentrations as well as under strictly anaerobic conditions. Besides, they can generate unwanted products such as xylitol [8]. From an economical perspective, along with the purity of the produced bioethanol, the use of liquid waste from distillation, which contains high concentrations of xylose and arabinose, applying pentose-fermenting yeasts or other potential microorganisms should be investigated in further study.

The uncompleted use of glucose in SSF might be related to stress conditions such as temperature, osmotic pressure, and inhibitor formation, which can inhibit the growth of yeasts and their ethanol production capacity [34]. Thermotolerant *S. cerevisiae* strains have been widely employed for bioethanol production from lignocellulosic biomasses via SSF. For example, Mendes et al. [38] reported that *S. cerevisiae* strain ATCC 26602 produced 41.7 g/L of ethanol, with a theoretical ethanol yield of 49.8% at 38 °C, when primary sludge was used as substrate. Da Silva et al. [39] studied ethanol production at 40 °C, using the *S. cerevisiae* strain CAT-1 and carnauba straw as substrate; ethanol concentration and theoretical yield were 5.47 g/L and 54.81%, respectively. Regarding this study, the bioethanol production by strain TC-5 at 42 °C was not significantly difference compared with that at 35–40 °C and of previous reports. In this sense, the thermotolerant strain TC-5 is a candidate yeast strain for bioethanol production under elevated temperature via SSF.

Ethanol production from various types of lignocellulosic biomass via the pre-SSF process has previously been investigated. For example, Gladis et al. [40] reported that bioethanol production from corn stover using baker’s yeast. Prehydrolysis was performed at 50 °C for 24 h, and after the prehydrolysis step, the glucose concentration was 111.5 g/L. Subsequently, the temperature was adjusted to 35 °C for yeast fermentation. An ethanol concentration and a theoretical ethanol yield of 53.5 g/L and 75% were attained, respectively. Öhgren et al. [41] reported that an ethanol concentration and theoretical ethanol yield of 26.4 g/L and 75%, obtained from corn stover by using baker’s yeast. Prehydrolysis was performed at 55 °C for 16 h, followed by SSF at 30 °C. Pre-SSF is one type of SSF process that provides the short period needed for lignocellulose material to be partly hydrolyzed before fermentation. This way, the prepared fermentable sugars in the fermentation medium can readily be used by the yeast. This process enables the use of higher temperatures during the initial enzymatic hydrolysis, potentially increasing enzymatic activity. Another advantage of this process is the reduction of the ethanol production time, potentially increasing the overall ethanol productivity [41].

Regarding the overall process time, to obtain the highest ethanol concentrations of SHF, pre-SSF, and SSF processes at 40 °C, periods of 168, 96, and 72 h, respectively, were needed. The bioethanol production via SSF and pre-SSF was not significantly different, whereas the maximum bioethanol productivity was obtained via SSF. In this sense, single-step SSF is suitable for bioethanol production from CRC at 40 °C because of the short fermentation and processing periods and the high ethanol productivity.

The ethanol production from various types of feedstocks via SSF at different solid loadings has been investigated. For example, Qin et al. [42] studied the effect of solid loading (6, 7, and 9%, *w*/*v*) on the bioethanol production from ethylenediamine-pretreated corn stover at 34 °C. The results showed that ethanol yield decreased with an increase in solid loading, and the authors suggested that this is caused by the lack of enzyme activity, cell viability, or other unknown factors. In addition, the challenges in bioethanol production via SSF at high solid concentrations are high viscosity and high energy consumption [43]. A high substrate concentration also results in a high content of inhibitors, impeding ethanol concentration and yield. These issues can be solved by using fed-batch fermentation [37,44].

Fed-batch SSF is a combination of batch and continuous modes by the periodical addition of the substrate into the fermentation medium and has been used to overcome substrate inhibition in batch SSF [37,40]. Ethanol production from various types of feedstocks via fed-batch SSF has been reported. For example, Gao et al. [37] reported bioethanol production from sugarcane bagasse with 33% (*w*/*v*) solid loading via fed-batch SSF. The highest ethanol concentration of 76 g/L, with a theoretical ethanol yield of 66%, was obtained. Fed-batch SSF using 20% (*w*/*v*) corn stover has been studied by Gladis et al. [40], and an ethanol concentration and a theoretical ethanol yield of 58 g/L and 81% were obtained. In this study, bioethanol production via fed-batch SSF with 12.5% (*w*/*v*) CRC solid loading at 40 °C provided the highest ethanol yield compared with other processes (Table 1). Therefore, fed-batch SSF at 12.5% (*w*/*v*) solid loading was also simulated for bioethanol production in a bioreactor.

The ethanol concentration in the bioreactor was lower than that obtained from via the 100-mL laboratory bottle (Table 1). This result is in agreement with previous findings. For example, Orrego et al. [45] found that the ethanol concentration attained from a 5-L bioreactor was lower than that obtained via a 250-mL Erlenmeyer flask. Dwiarti et al. [46] also found that the ethanol production in 2-L Erlenmeyer flasks was decreased compared with that of 200-mL Erlenmeyer flasks (11.80 to 8 g/L). Most likely, this can be explained by insufficient oxygen availability, mass, and heat transfer coefficients, absence of free water, accumulation of unused sugar (xylose and arabinose), viscosity, and mixing capacity [45,46,47].

During alkali pretreatment, the intermolecular ester bonds between lignin and hemicellulose are saponified, and the hemicellulose and lignin are dissolved in the alkali solution [48]. Therefore, the cellulose contents in KOH-treated corncob increased to 64.46%, whereas the hemicellulose and lignin contents decreased to 31.27 and 2.52%, respectively. After hydrolysis by endo-xylanase, 115 g of XOs and 515 g of CRC residue were obtained. Endo-xylanase is the key enzyme for hemicellulose hydrolysis and can randomly break down xylan, the main hemicellulose found in corncob, to produce XOs [49]; in treated corncob, the cellulose remained. Consequently, the cellulose content in CRC residue was relatively high (75.69%), and the hemicellulose content decreased to 14.53% (Figure 4). Hence, CRC residue with a high cellulose content could be a potential substrate for bioethanol production.

Table 2 shows the comparison of bioethanol production by different strains of *S. cerevisiae* from various types of lignocellulosic biomass via SSF at higher temperatures. Saravanikumar and Kathiresan [50], in their studies on sawdust, reported that sawdust hydrolysate was produced by consecutive acid treatment and enzymatic hydrolysis, using cellulase from *Trichoderma estonicum* SKS1. Bioethanol was produced via SHF by *S. cerevisiae* JN387604 at 36.5 °C, obtaining an ethanol concentration of 55.2 g/L and a theoretical yield of 85.6% at a fermentation period of 102 h. Recently, waste jasmine flower has been used as substrate for bioethanol production. The biomass was first treated using alkaline solutions and temperature, followed by enzymatic hydrolysis using cellulase for 24 h to obtain fermentable sugars. Then, bioethanol was produced using free cells of *S. cerevisiae* TISTR5020 in a 2-L bioreactor at 30–35 °C via SHF. Ethanol concentration, productivity, and yield were 14.39 g/L, 0.029 g/L/h, and 0.029 g_EtOH_/g_biomass_, obtained within 120 h [51].

With regard to fed-batch SSF (Table 3), another bioethanol production process has been reported by Gao et al. [37], who investigated bioethanol production from sugarcane bagasse via fed-batch SSF by using *S. cerevisiae* Y-2034 and commercial cellulase (Novozymes A/S). Fermentation was carried out in 250-mL Erlenmeyer flasks (100 mL of reaction volume) at 37 °C, an ethanol concentration and theoretical ethanol yield of 46.13 g/L and 70.06%, respectively, were obtained. In another study, bioethanol production from empty palm fruit bunch fiber in a 5-L stirring bioreactor (1 L of reaction volume) via fed-batch SSF has been investigated by Park et al. [53]. The strain *S. cerevisiae* L3262a was employed to produce ethanol at 30 °C, using fermentation medium with 10 g/L of yeast extract and 20 g/L of peptone. Under optimal conditions, an ethanol concentration of 62.5 g/L was obtained, with a theoretical yield of 70.6%. In a study by Tareen et al. [52], an oil palm trunk was treated by steam explosion and alkali extraction, followed by SSF. Fermentation was carried out in 500-mL flasks containing 300 mL of SSF production medium (10% treated oil palm trunk, 20 g/L of peptone, and 10 g/L of yeast extract). Two types of commercial cellulase, namely Celluclast 1.5 L and Novozymes 188 were employed in the SSF process, which was carried out at 40 °C using *S. cerevisiae* SC90. The highest ethanol concentration was 44.25 g/L, with a productivity of 0.45 g/L/h.

In the present study, ethanol concentration, ethanol yield, and theoretical ethanol yield values of 31.96 g/L (143 g/kg_raw corncob_), 0.256 g_EtOH_/g_CRC_, and 78.20%, respectively, were achieved by fed-batch SSF in a 5-L bioreactor. Ethanol concentration and productivity were not only comparable to those obtained in previous studies, but the cost of the SSF production medium used in this study was also remarkably low. Only 1 g/L of yeast extract was used, and inexpensive inorganic nutrients ((NH_4_)_2_SO_4_, NH_4_H_2_PO_4_, and MgSO_4_·7H_2_O) were supplemented. Alternatively, *S. cerevisiae* TC-5 could grow and effectively produce bioethanol even under low nutrient conditions. The cost of these supplemental ingredients is a critical issue for industrial processes or commercial ethanol plants [54]. Yeast extract alone is estimated to account for approximately 20% of the raw material costs [55]. When medium with urea 3 g/L was used to produce bioethanol from carob waste, compared with the use of yeast extract-peptone medium (10 g/L of yeast extract and 20 g/L of peptone), the production costs could be reduced by up to 50% [56]. Another aspect for ethanol production is the minimization of the overall process time. Conventional SHF is considered a time-consuming process [57]. In this study, the overall process time of the up-scaled ethanol production, using thermotolerant *S. cerevisiae* TC-5, via SSF was only 144 h. In this sense, the newly isolated *S. cerevisiae* TC-5 might be an effective thermotolerant yeast strain suitable for bioethanol production from lignocellulosic material, minimizing processing time, and production costs. Regarding these concerns, this ethanol production procedure using *S. cerevisiae* TC-5 is effective, practical, and competitive on a cost basis.

## 5. Conclusions

This study shows that CRC residue can be used as a potential second-generation substrate for bioethanol production because of its high cellulose content. The isolated thermotolerant strain *S. cerevisiae* TC-5 could grow and produce bioethanol at 40 °C and required less supplemented nutrients and minerals. In addition, strain TC-5 could be employed in either batch SSF or fed-batch SSF at bioreactor scale. These characteristics make it an attractive thermotolerant yeast strain suitable for use in industrial bioethanol production from various lignocellulosic biomasses.

## Figures and Tables

**Table 1:** Ethanol production from CRC via batch and fed-batch simultaneous saccharification and fermentation (SSF) at different cellulose-rich corncob (CRC) solid loadings by the thermotolerant *S. cerevisiae* TC-5 at 40 °C.

Processes	CRC Loading (%, *w*/*v*)	Time (h)	C_EtOH_ * (g/L)	Y_p/s_ **		Q_P_ *** (g/L/h)	Y **** (%)
				g_EtOH_/g_glucose_	g_EtOH_/g_CRC_		
**100-mL laboratory Bottle**							
Batch	7.5	72	20.92 ± 0.16 ^Gd^	0.436 ± 0.003 ^Cb^	0.269 ± 0.002 ^Cb^	0.291 ± 0.002 ^Ab^	80.26 ± 0.63 ^Cb^
	10	120	28.90 ± 0.17 ^Fc^	0.452 ± 0.003 ^Ba^	0.289 ± 0.001 ^Ba^	0.241 ± 0.001 ^Da^	88.37 ± 0.52 ^Ba^
	12.5	144	35.91 ± 0.30 ^Ca^	0.449 ± 0.004 ^Ba^	0.288 ± 0.002 ^Ba^	0.249 ± 0.002 ^Ca^	87.86 ± 0.73 ^Ba^
	15	168	34.90 ± 0.01 ^Db^	0.364 ± 0.000 ^Fc^	0.233 ± 0.000 ^Fc^	0.208 ± 0.000 ^Fc^	71.13 ± 0.01 ^Fc^
Fed-batch	10	120	28.70 ± 0.11 ^Fb^	0.448 ± 0.002 ^Bb^	0.287 ± 0.001 ^Bb^	0.239 ± 0.001 ^Db^	87.74 ± 0.32 ^Bb^
	12.5	144	38.23 ± 0.19 ^Aa^	0.478 ± 0.002 ^Aa^	0.306 ± 0.001 ^Aa^	0.265 ± 0.001 ^Ba^	93.51 ± 0.47 ^Aa^
	15	168	37.55 ± 0.35 ^Bb^	0.391 ± 0.004 ^Ec^	0.251 ± 0.002 ^Ec^	0.224 ± 0.002 ^Ec^	76.56 ± 0.70 ^Ec^
**5-L bioreactor**							
Fed-batch	12.5	144	31.96 ± 0.78 ^E^	0.400 ± 0.000 ^D^	0.256 ± 0.001 ^D^	0.222 ± 0.001 ^E^	78.20 ± 0.19 ^D^

Note: values are presented as mean ± standard deviation. Data with the same superscript (capital letter) in the same column are not significantly different at *p* ≤ 0.05 (different kinetic values were compared among all fermentation processes and CRC loadings). Data with the same superscript (small letter) are not significantly different at *p* ≤ 0.05 (different kinetic values were compared among all fermentation processes and CRC loadings). The level of significance was tested by Duncan’s multiple range test at *p* ≤ 0.05. * C_EtOH_: ethanol concentration; ** Y_p/s_: ethanol yield; *** Q_P_: ethanol productivity; **** Y: theoretical ethanol yield.

**Table 2:** Comparison of bioethanol production from *S. cerevisiae* using lignocellulosic biomass via simultaneous saccharification and fermentation (SSF).

Strain	Solid Loading; Scale	Fermentation Temperature (°C)	Substrate	C_EtOH_ * (g/L)	Y_p/s_ ** g_EtOH_/g_substrate_	Q_P_ *** (g/L/h)	Y **** (%)	Reference
*S. cerevisiae* mbc2	9% (*w*/*w*) glucan; Flask-scale	42	Silvergrass (*Miscanthus* sp.)	15.30	N/A	0.32	90.10	Cha et al. [12]
*S. cerevisiae* TJ14	5% (*w*/*v*) cellulose; Flask-scale	42	Paper sludge	11.80	N/A	0.12	80.00	Dwiarti et al. [46]
*S. cerevisiae* SC90	10% (*w*/*v*); Flask-scale	40	Oil palm trunk	44.25	0.443	0.46	90.34	Tareen et al. [52]
*S. cerevisiae* TC-5	12.5% (*w*/*v*); Flask-scale	40	Cellulose-rich corncob residue	35.91	0.251	0.25	87.86	This study

Note: N/A: not available; * C_EtOH_: ethanol concentration; ** Y_p/s_: ethanol yield; *** Q_P_: ethanol productivity; **** Y: theoretical ethanol yield.

**Table 3:** Comparison of bioethanol production from *S. cerevisiae* using lignocellulosic biomass via fed-batch simultaneous saccharification and fermentation (fed-batch SSF).

Strain	Solid Loading; Scale	Fermentation Temperature (°C)	Substrate	C_EtOH_ * (g/L)	Y_p/s_ ** g_EtOH_/g_substrate_	Q_P_ *** (g/L/h)	Y **** (%)	Reference
*S. cerevisiae* Y-2034	19% (*w*/*v*); Flask-scale	37	Sugarcane bagasse	46.13	0.243	0.64	70.06	Gao et al. [37]
*S. cerevisiae* L2524a	30% (*w*/*v*); Bioreactor-scale	30	Empty palm fruit bunch fibers	62.5	0.208	0.66	70.60	Park et al. [53]
*S. cerevisiae* TC-5	12.5% (*w*/*v*); Bioreactor-scale	40	Cellulose-rich corncob residue	31.96	0.256	0.22	78.20	This study

Note: N/A: not available; * C_EtOH_: ethanol concentration; ** Yp/s: ethanol yield; *** QP: ethanol productivity; **** Y: theoretical ethanol yield.

## Data Availability

The 26S rDNA sequences data obtained in this work have been deposited in NCBI and are available on request from the corresponding author (thanongsak.c@cmu.ac.th or thachaiyaso@hotmail.com (accessed on 9 July 2021)).

## Supplementary Materials

The following are available online at https://www.mdpi.com/article/10.3390/jof7070547/s1, Table S1: Comparison of ethanol concentration, ethanol yield, ethanol productivity, and theoretical ethanol yield produced by commercial *S. cerevisiae* (control) and the thermotolerant *S. cerevisiae* TC-5 and via separate hydrolysis and fermentation (SHF), simultaneous saccharification and fermentation (SSF), and prehydrolysis-simultaneous saccharification and fermentation (pre-SSF) at 35, 37, 40, and 42 °C at 72 h of fermentation time. Figure S1: Phylogenetic tree of the thermotolerant *S. cerevisiae* TC-5, commercial *S. cerevisiae*, and the related species in GenBank database. Figure S2: Time course of ethanol production from cellulose-rich corncob hydrolysate by commercial *S. cerevisiae* via separate hydrolysis and fermentation (SHF) process at 35 (a), 37 (b), 40 (c), and 42 °C (d). Figure S3: Time course of ethanol production from cellulose-rich corncob hydrolysate by *S. cerevisiae* TC-5 via separate hydrolysis and fermentation (SHF) process at 35 (a), 37 (b), 40 (c), and 42 °C (d). Figure S4: Time course of ethanol production from cellulose-rich corncob residue by commercial *S. cerevisiae* via simultaneous saccharification and fermentation (SSF) process at 35 (a), 37 (b), 40 (c), and 42 °C (d). Figure S5: Time course of ethanol production from cellulose-rich corncob residue by *S. cerevisiae* TC-5 via simultaneous saccharification and fermentation (SSF) process at 35 (a), 37 (b), 40 (c), and 42 °C (d). Figure S6: Time course of ethanol production from cellulose-rich corncob residue by commercial *S. cerevisiae* via prehydrolysis-simultaneous saccharification and fermentation (pre-SSF) process at 35 (a), 37 (b), 40 (c), and 42 °C (d). Figure S7: Time course of ethanol production from cellulose-rich corncob residue by *S. cerevisiae* TC-5 via prehydrolysis-simultaneous saccharification and fermentation (pre-SSF) process at 35 (a), 37 (b), 40 (c), and 42 °C (d).
