# Supplementary material for: Bioethanol Production from Cellulose-Rich Corncob Residue by the Thermotolerant Saccharomyces cerevisiae TC-5
Source: J Fungi (Basel). 2021 Jul 9;7(7):547. doi: 10.3390/jof7070547 (PMC8305858; doi:10.3390/jof7070547)
Supplement: Supplementary file 1 [file jof-07-00547-s001.zip › jof-1281857-supplementary.pdf]

Supplementary Data

Journal name: Journal of Fungi

Bioethanol Production from Cellulose-rich Corncob Residue by the Thermotolerant *Saccharomyces cerevisiae* TC-5

Pinpanit Boonchuay[1], Charin Techapun[1], Noppol Leksawasdi[2], Phisit Seesuriyachan[1], Prasert Hanmoungjai[1], Masanori Watanabe[3], Siraprapa Srisupa[1], and Thanongsak Chaiyaso[1*]

[1] Division of Biotechnology, Faculty of Agro-Industry, Chiang Mai University, Chiang Mai 50100, Thailand

[2] Division of Food Engineering, Faculty of Agro-Industry, Chiang Mai University, Chiang Mai 50100, Thailand

[3] Department of Food, Life and Environmental Sciences, Faculty of Agriculture, Yamagata University, Tsuruoka, Yamagata 9978555, Japan

[*] Corresponding author at: Division of Biotechnology, Faculty of Agro-Industry, Chiang Mai University, Chiang Mai 50100, Thailand

Tel: +66 5394 8262, Fax: +66 5394 8219

✉ thachaiyaso@hotmail.com; thanongsak.c@cmu.ac.th (T. Chaiyaso)

Table S1 Comparison of ethanol concentration, ethanol yield, ethanol productivity, and theoretical ethanol yield produced by commercial *S. cerevisiae* (control) and the thermotolerant *S. cerevisiae* TC-5 via separate hydrolysis and fermentation (SHF), simultaneous saccharification and fermentation (SSF), and prehydrolysis-simultaneous saccharification and fermentation (pre-SSF) at 35, 37, 40, and 42°C at 72 h of fermentation time.

Temperature (°C)	Strains	C_{EtOH}* (g/L)	$Y_{p/s}$** ($g_{EtOH}/g_{glucose}$)	Q_P*** (g/L/h)	Y**** (%)
**Separate hydrolysis and fermentation (SHF) process**					
35	Commercial *S. cerevisiae*	20.59±0.35	0.378±0.000	0.123±0.001	79.01±0.32
	S. cerevisiae TC-5	20.33±0.92	0.373±0.021	0.121±0.005	78.01±3.33
37	Commercial *S. cerevisiae*	19.09±0.11	0.351±0.000	0.114±0.001	73.25±0.41
	S. cerevisiae TC-5	21.64±1.06	0.397±0.022	0.129±0.006	83.04±3.68
40	Commercial *S. cerevisiae*	12.42±1.08	0.228±0.021	0.074±0.006	47.66±3.93
	S. cerevisiae TC-5	20.50±0.68	0.376±0.014	0.122±0.004	78.66±2.47
42	Commercial *S. cerevisiae*	9.81±0.44	0.180±0.014	0.058±0.002	37.64±1.61
	S. cerevisiae TC-5	18.39±0.35	0.338±0.007	0.109±0.002	70.57±1.25
**Simultaneous saccharification and fermentation (SSF) process**					
35	Commercial *S. cerevisiae*	17.58±0.52	0.323±0.007	0.244±0.007	67.46±1.89
	S. cerevisiae TC-5	20.69±0.61	0.380±0.014	0.287±0.008	79.39±2.21

Temperature (°C)	Strains	C_{EtOH}* (g/L)	$Y_{p/s}$** ($g_{EtOH}/g_{glucose}$)	Q_P*** (g/L/h)	Y**** (%)
37	Commercial *S. cerevisiae*	16.58±0.04	0.304±0.000	0.230±0.001	63.62±0.13
	S. cerevisiae TC-5	20.73±0.25	0.381±0.000	0.288±0.004	79.54±0.93
40	Commercial *S. cerevisiae*	12.81±0.78	0.235±0.014	0.178±0.011	49.15±2.82
	S. cerevisiae TC-5	20.92±0.34	0.384±0.003	0.291±0.002	80.27±1.23
42	Commercial *S. cerevisiae*	7.45±0.40	0.137±0.007	0.103±0.006	28.59±1.46
	S. cerevisiae TC-5	20.02±0.37	0.368±0.007	0.278±0.006	76.82±1.33
**Prehydrolysis-simultaneous saccharification and fermentation (pre-SSF) process**					
35	Commercial *S. cerevisiae*	20.71±0.06	0.380±0.000	0.216±0.011	79.47±0.23
	S. cerevisiae TC-5	20.10±0.61	0.370±0.014	0.209±0.006	77.13±2.21
37	Commercial *S. cerevisiae*	20.19±1.04	0.370±0.014	0.211±0.011	77.49±3.76
	S. cerevisiae TC-5	20.13±0.04	0.370±0.000	0.210±0.007	77.24±0.16
40	Commercial *S. cerevisiae*	12.15±0.45	0.220±0.000	0.127±0.00	46.62±0.25
	S. cerevisiae TC-5	20.89±0.44	0.385±0.007	0.218±0.005	80.16±1.61
42	Commercial *S. cerevisiae*	8.24±0.01	0.150±0.000	0.086±0.000	31.62±0.05
	S. cerevisiae TC-5	19.91±0.22	0.365±0.007	0.207±0.002	76.40±0.80

Note: Values are presented as mean ± standard deviation. $^{*}C_{EtOH}$: ethanol concentration; $Y_{p/s}^{**}$: ethanol yield; Q_P^{***}: ethanol productivity; Y^{****}: theoretical ethanol yield.

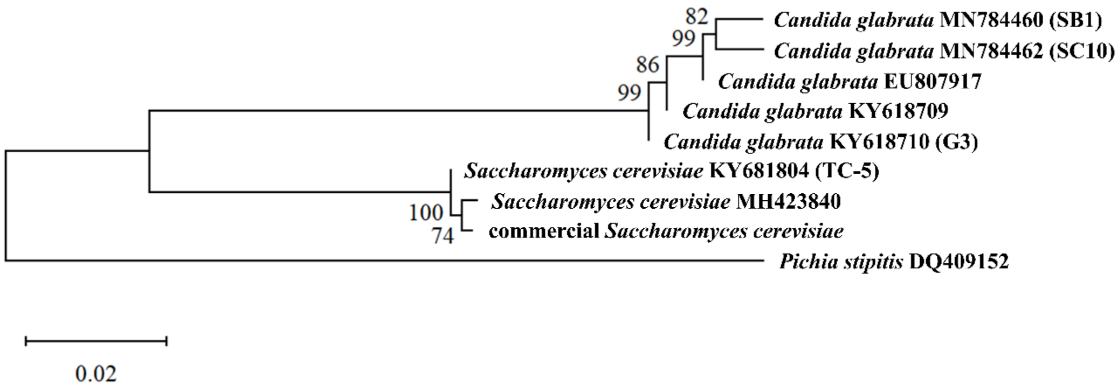

Figure S1 Phylogenetic tree of the *S. cerevisiae* (commercial strain, Danstil, Lallemand Inc., Denmark), thermotolerant *S. cerevisiae* TC-5, *C. glabrata* SB1, *C. glabrata* SC10, *C. glabrata* G3, and the related species in GenBank database.

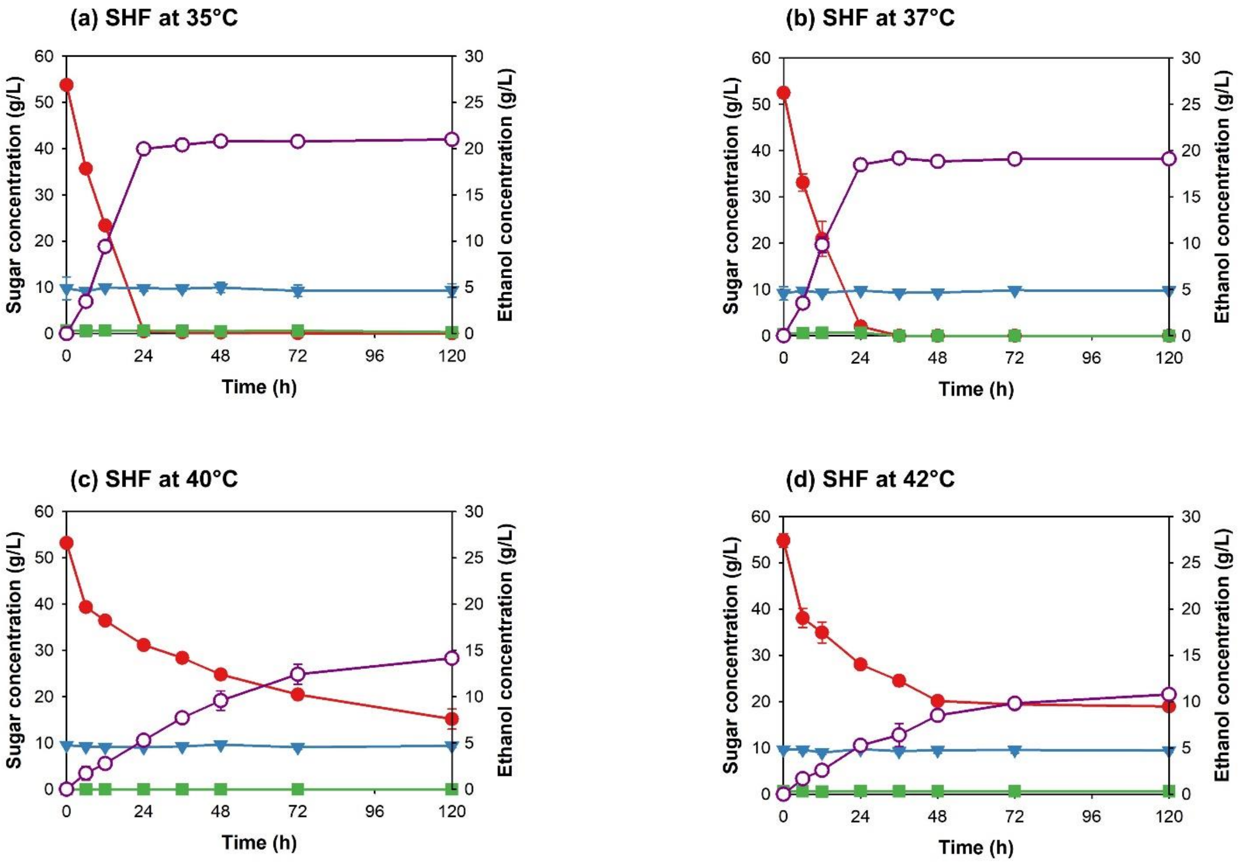

Figure S2 Time course of ethanol production from cellulose-rich corncob hydrolysate by commercial *S. cerevisiae* via separate hydrolysis and fermentation (SHF) process at 35 (a), 37 (b), 40 (c), and 42°C (d).

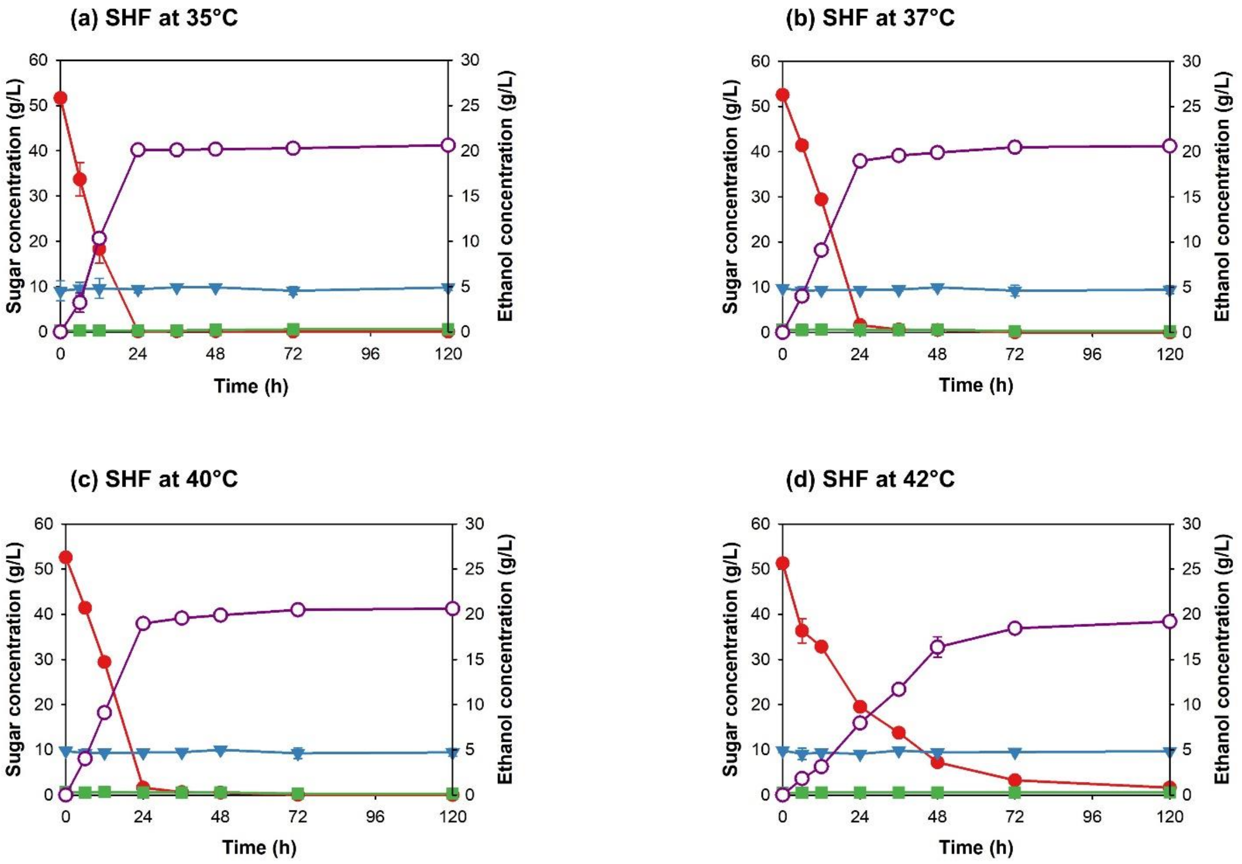

Figure S3 Time course of ethanol production from cellulose-rich corncob hydrolysate by *S. cerevisiae* TC-5 via separate hydrolysis and fermentation (SHF) process at 35 (a), 37 (b), 40 (c), and 42°C (d).

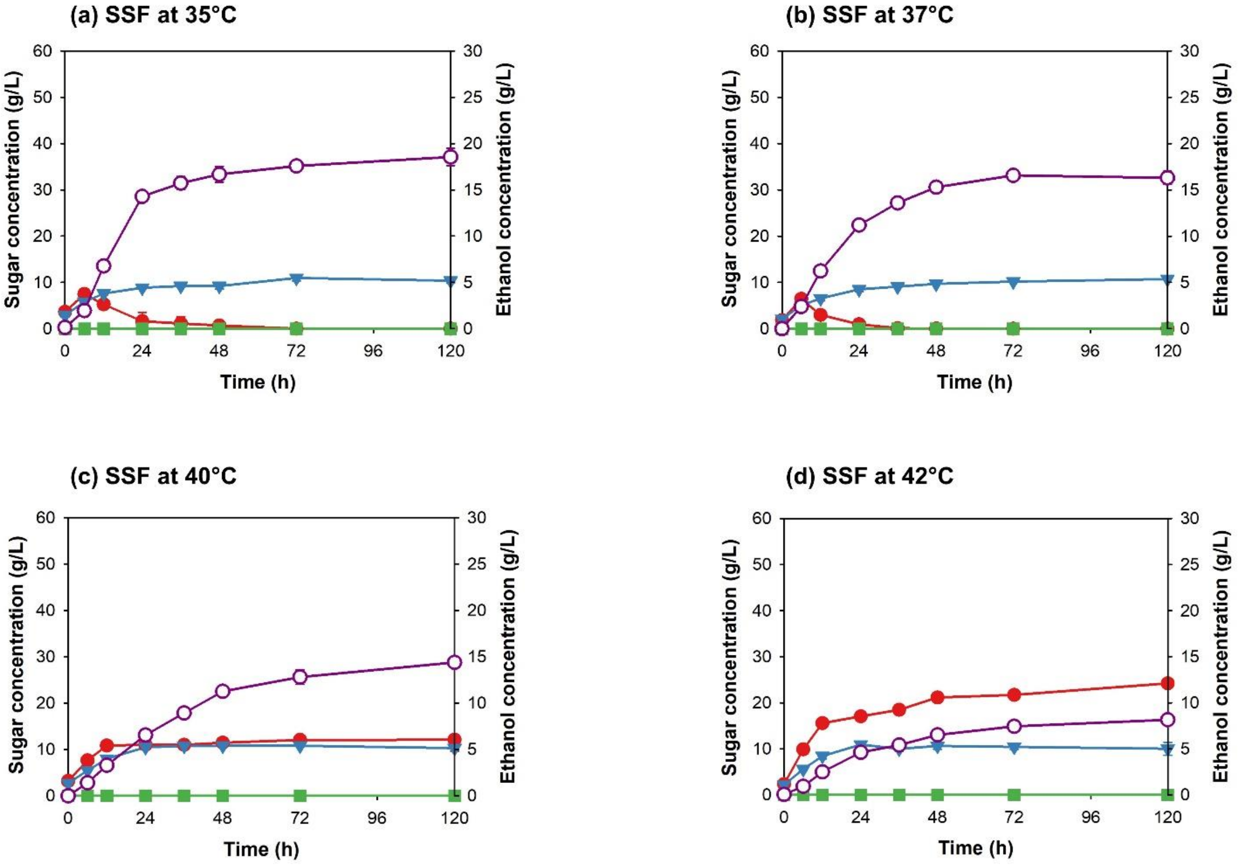

Figure S4 Time course of ethanol production from cellulose-rich corncob residue by commercial *S. cerevisiae* via simultaneous saccharification and fermentation (SSF) process at 35 (a), 37 (b), 40 (c), and 42°C (d).

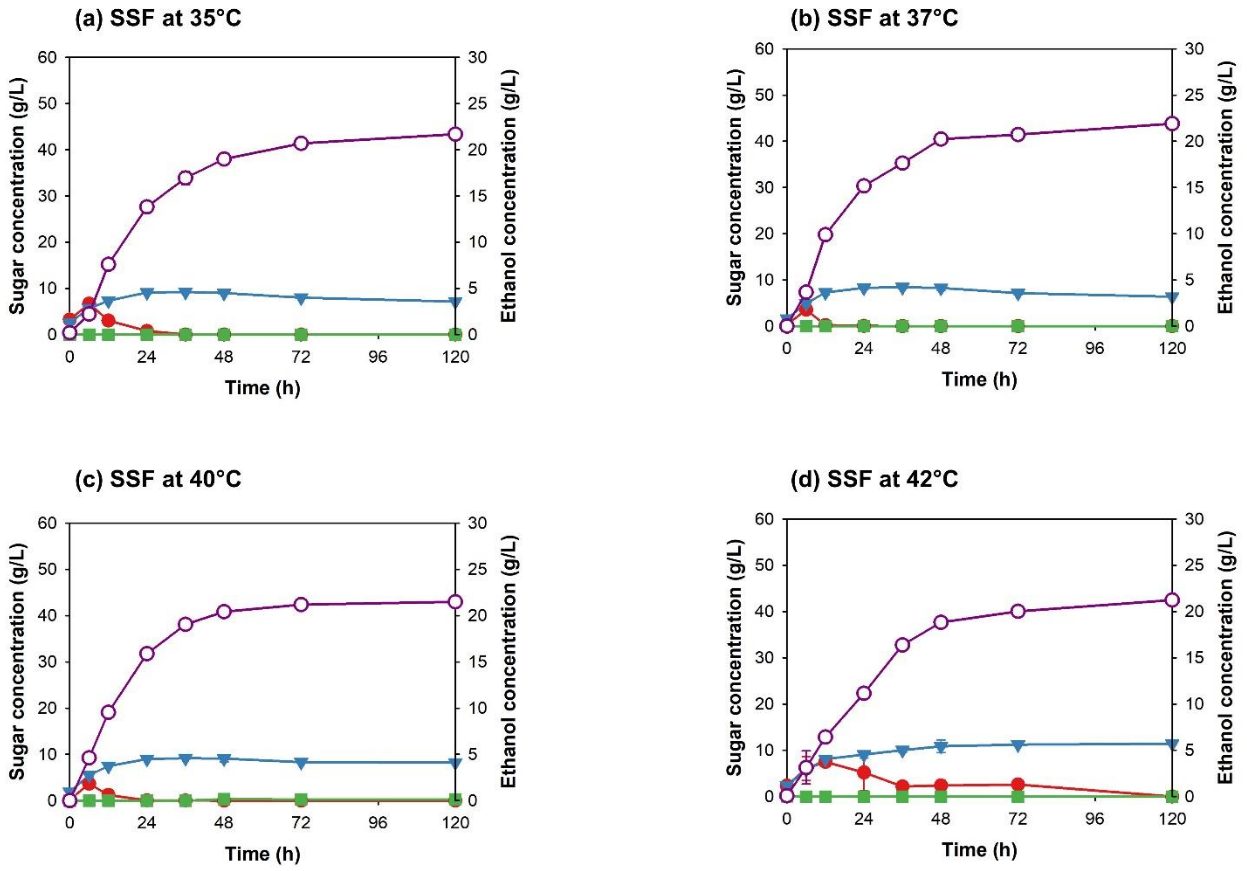

Figure S5 Time course of ethanol production from cellulose-rich corncob residue by *S. cerevisiae* TC-5 via simultaneous saccharification and fermentation (SSF) process at 35 (a), 37 (b), 40 (c), and 42°C (d).

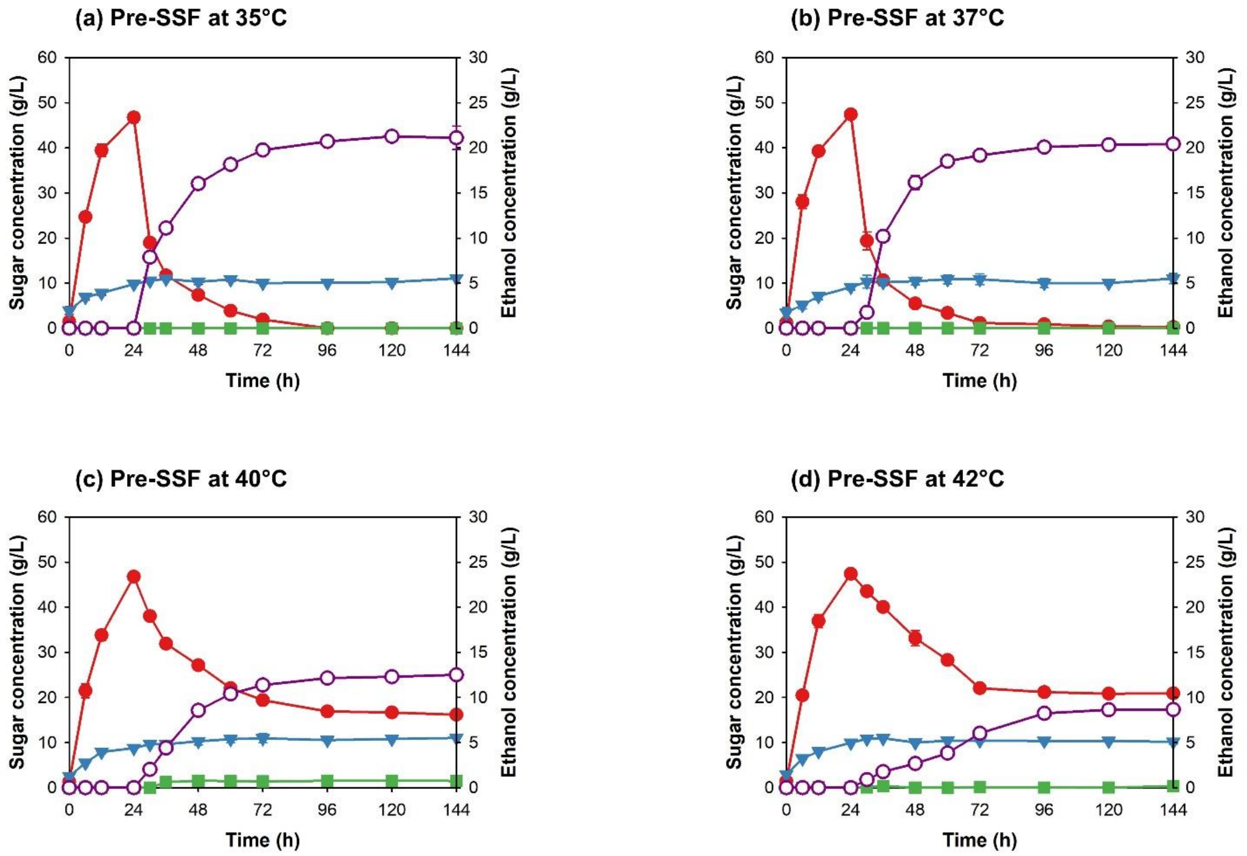

Figure S6 Time course of ethanol production from cellulose-rich corncob residue by commercial *S. cerevisiae* via prehydrolysis-simultaneous saccharification and fermentation (pre-SSF) process at 35 (a), 37 (b), 40 (c), and 42°C (d).

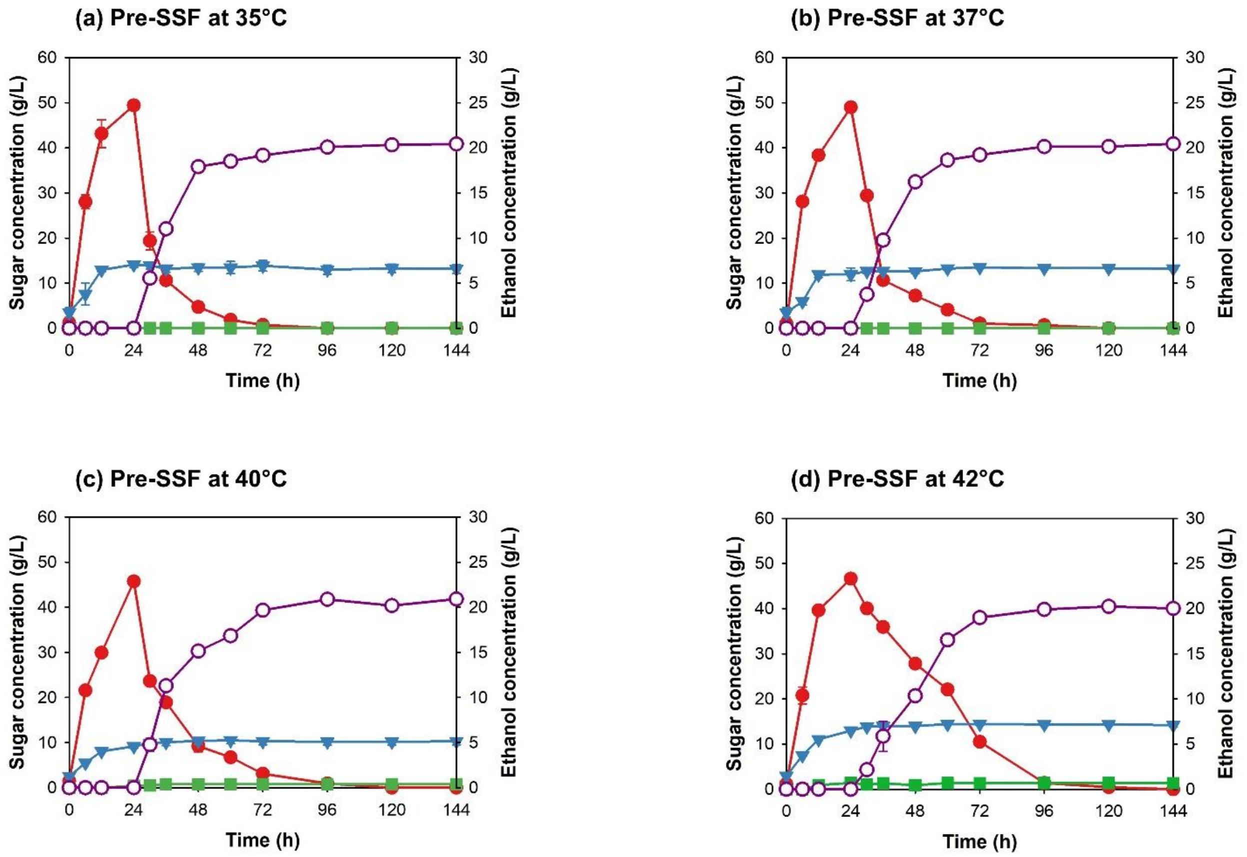

Figure S7 Time course of ethanol production from cellulose-rich corncob residue by *S. cerevisiae* TC-5 via prehydrolysis-simultaneous saccharification and fermentation (pre-SSF) process at 35 (a), 37 (b), 40 (c), and 42°C (d).
